# Middle mediastinal paraganglioma: A case report and review of the literature

**DOI:** 10.1097/MD.0000000000036327

**Published:** 2023-11-24

**Authors:** Shaopeng Xu, Gawei Hu, Jinchen Du, Linchong Ma, Lei Zou, Qingxin Li

**Affiliations:** a Department of General Thoracic Surgery, 940th Hospital of Joint Logistic Support Force of Chinese People’s Liberation Army, Lanzhou, China; b Ningxia Medical University, Yinchuan, China.

**Keywords:** case report, mediastinum, nonfunctional tumor, paraganglioma, treatment

## Abstract

**Rational:**

Paragangliomas are rare and can occur in many places throughout the body, but mediastinal paragangliomas are even rarer, accounting for less than 0.3% of mediastinal masses. Extremely susceptible to misdiagnosis and mistreatment, which may lead to the death of the patient.

**Patient concerns:**

We report a case of a giant paraganglioma of the middle mediastinum. A 40-year-old woman was admitted to the hospital with a rib fracture and a chest computed tomography suggesting a giant occupying tumor in the middle mediastinum.

**Diagnosis:**

Immunohistochemistry revealed positive for S100 fraction and Syn, focally positive for CgA, while negative for CKp and succinate dehydrogenase complex iron sulfur subunit B gene, and Ki67index ≈ 5%. The imaging and immunohistochemical features suggested a final diagnosis of Paragangliomas.

**Interventions:**

This patient underwent lateral open heart surgery to remove a mediastinal mass.

**Outcomes:**

One month after being discharged, the patient was contacted by phone for a follow-up visit and reported feeling OK. Unfortunately, as of the date of submission, the patient did not come to our hospital for review.

**Lessons:**

Mediastinal paraganglioma as a rare and potentially malignant tumor susceptible to misdiagnosis and mistreatment. Organ pathology examination is the gold standard for diagnosis, and surgery is an important treatment method. A clear diagnosis and thorough preoperative examination are important guarantees for the success of surgery.

## 1. Introduction

Paragangliomas are neuroendocrine tumors derived from neural crest cells, occurring in about 2 to 5 cases per million,^[1]^ and can occur in various parts of the body, including the head and neck, lungs, mediastinum, liver, retroperitoneum, and small intestine, with the head and neck being the most common, accounting for about 69% of cases,^[2]^ while those in the mediastinum are rarer. Here, we report a case of a giant paraganglioma of the middle mediastinum that was treated surgically and discharged from the hospital.

## 2. Case report

**Figure 1.**
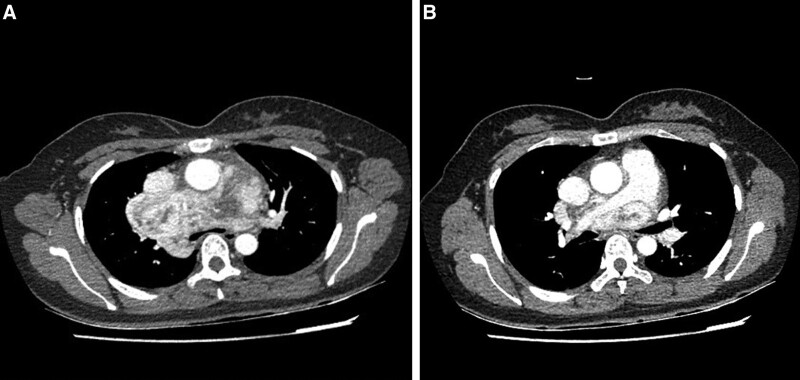
Enhanced CT of the chest indicates that the tumor is rich in blood supply. (A) The tracheal ridge is deformed by compression. (B) The tumor tissue wraps around the aorta and pulmonary artery. CT = computed tomography.

A 40-year-old female presented to our hospital on February 20, 2023, with a mediastinal mass found on chest CT for rib fracture. The patient had no ptosis; no headache or dizziness; no chest tightness or shortness of breath; no chest pain or hemoptysis, and no history of palpitations, panic and facial flushing. The past medical history was not specific. Her temperature was 36.7ºC, heart rate was 82 beats/min, respiratory rate was 18 breaths/min, the blood pressure was 102/67 mm Hg. No positive signs were detected on physical examination. Enhanced CT of the chest suggests: mediastinal widening, and a large morphologically irregular and abnormally enhancing soft tissue occupancy with lobulated growth is seen in the mediastinum during the enhanced arterial phase. Patchy and nodular enhancement is seen in the arterial phase of the lesion, and contrast fills centripetally in the venous phase with an abundant blood supply. The size of the lesion is about 11.5 cm × 6.1 cm × 8.6 cm, up to the entrance of the thorax and down to the upper edge of the left atrium. The lesion is close to large blood vessels and trachea, the trachea and the left and right main bronchi are compressed, consider a tumor lesion (Fig. 1). Ultrasound of superficial organs and whole-body bone scan showed no significant abnormalities; laboratory tests such as routine blood, liver function, kidney function, urine epinephrine, norepinephrine, methoxyepinephrine and methoxynorepinephrine showed no abnormalities.

**Figure 2.**
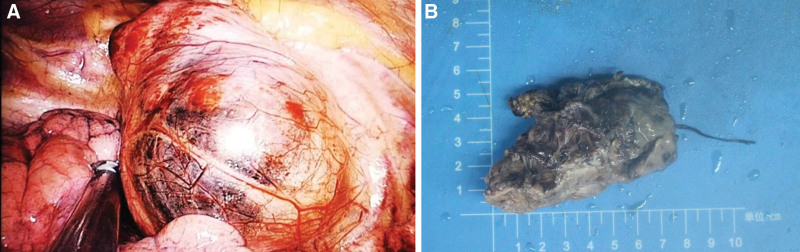
(A) Thoracoscopic surface vascular varices in mediastinal masses. (B) Formalin-fixed mediastinal masses.

**Figure 3.**
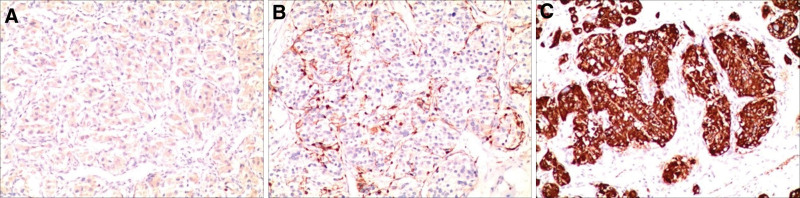
(A) Immunohistochemical staining CgA(+/−) (×100). (B) Immunohistochemical staining S100 fraction(+) (×100). (C) Immunohistochemical staining Syn(+) (×100).

The surgery took 4 cm between the 4th rib and the right mid-axillary line for the lumpectomy observation and operation hole. Thoracoscopic examination revealed a tumor with a soft texture and a rich blood supply that measured about 12 × 9 cm and was located in the middle of the mediastinum. The tumor’s surface was covered in varicose blood vessels (Fig. 2). The tumor was growing along the main pulmonary artery’s interstitial space. Considering that it was difficult to remove the tumor completely under thoracoscopy, the surgical incision was extended to 12 cm and the tumor was finally removed completely. The total amount of bleeding in the middle was 2400 mL, 1100 mL of suspended red blood cells and 1200 mL of plasma were transfused. Postoperative pathology suggested that the tumor cells were arranged in a glandular vesicular or mass-like pattern with interstitial enriched vascular and fibrous tissue. Immunohistochemical results: CKp(−), CgA(+/−), Ki67index ≈ 5%, S100 fraction(+), succinate dehydrogenase complex iron sulfur subunit B gene(−), and Syn(+) (Fig. 3).

One month after being discharged, the patient was contacted by phone for a follow-up visit and reported feeling OK. Unfortunately, as of the date of submission, the patient did not come to our hospital for review.

## 3. Discussion

Patients with paragangliomas often have ages between 40 and 50, regardless of gender,^[3]^ and can be clinically classified into functional and nonfunctional tumors; about 50% to 80% of paragangliomas are nonfunctional tumors,^[4]^ and patients are mostly asymptomatic and discovered by chance; they can also develop corresponding clinical symptoms, such as cough, dyspnea, and other discomforts due to tumor compression of surrounding tissues. It is sufficiently large at the time of clinical findings. Functional paragangliomas have a endocrine function and can secrete catecholamines such as epinephrine, norepinephrine, and dopamine. This causes hypertension, excessive sweating, headache, and palpitations. The concentration of catecholamines in blood and metabolites of catecholamines in urine, such as methoxyprenaline and methoxynorepinephrine, provide further diagnosis.

Mediastinal paragangliomas is extremely rare clinically, accounting for approximately 1% to 2% of paragangliomas and less than 0.3% of mediastinal masses. The first case of paraganglioma was reported by Lattes in 1950.^[5,6]^ Takashima et al^[7]^ divided them into main pulmonary paragangliomas occurring in the anterior or middle mediastinum and main sympathetic paragangliomas occurring in the posterior mediastinum. Nonfunctional tumors of pulmonary paragangliomas are more common than functional tumors and occur more often in people over 40 years of age. Paragangliomas are difficult to differentiate from other mediastinal tumors, such as malignant lymphomas and angiosarcomas, on imaging and often rely on histological examination and immunohistochemistry. Paragangliomas often express chromophobic proteins, synaptophysin, neuron-specific enolase, CD56 and supporting cell S100 protein. In the present case, the tumor expressed supporting cell S100 protein, supporting the diagnosis of mediastinal ganglioneuroma.

Paragangliomas are slow growing and about 90% are benign,^[3]^ but they have malignant potential, including local invasiveness and distant metastases, with long-term survival rates of up to 84% in patients with complete surgical resection and 50% in patients with partial resection^[8]^; therefore, surgery is considered the preferred modality for the treatment of chemoreceptor tumors. In addition, vital organs including the heart, the aorta, the pulmonary artery, esophagus, and trachea are located in the mediastinum, and mediastinal paraganglioma usually requires median sternotomy under extracorporeal circulation for complete resection of the lesion in order to facilitate the operation and reduce the operative time, intraoperative bleeding, and postoperative complications.^[6]^ For paragangliomas with an excessive blood supply, preoperative vascular embolization is also possible to stop the blood flow to the tumor, shrink the tumor volume, further reduce intraoperative bleeding and intraoperative hemodynamic fluctuations, and selective vascular embolization can also slow down the development of the tumor for patients who are difficult to remove surgically or difficult to remove completely. Shakir et al^[9]^ in a case of 4.3 cm × 5.7 cm × 8.7 cm anterior mediastinal paraganglioma, preoperative selective embolization of the main blood supply vessels of the tumor followed by median sternotomy for tumor resection did not show significant intraoperative blood loss and extracorporeal circulation was not performed. Thoracoscopic or minimally invasive robotic-assisted resection may also be considered if the tumor is detected early clinically and is small or favorably located.^[10]^

The efficacy of chemotherapy in paraganglioma is uncertain, and it was previously thought that chemotherapy was insensitive to paraganglioma due to drug resistance. However, some patients can be completely cured after combination chemotherapy with cyclophosphamide, vincristine and dacarbazine, and stabilization of multiple metastatic paragangliomas of the bladder with octreotide in combination with cyclophosphamide, vincristine and dacarbazine chemotherapy has also been reported.^[11,12]^ The temozolomide is also a classic drug for chemotherapy of paraganglioma. Therefore, it has been suggested that chemotherapy can be used as an adjuvant treatment for malignant paragangliomas. It was previously thought that radiation therapy was equally insensitive to paraganglioma. However, it has been documented that in patients with advanced or unresectable metastatic paraganglioma, radiotherapy is effective in improving symptoms and controlling tumor growth, and higher radiation doses are associated with lower tumor recurrence.^[13]^ In addition, patients with succinate dehydrogenase (SDH)-positive paragangliomas can achieve partial remission after treatment with tyrosine kinase inhibitors^[14]^; Sesti et al^[15]^ reported that the multitargeted RTK inhibitor Sunitinib may be a viable treatment option for patients with inoperable paragangliomas. Overall, paragangliomas are clinically rare and the specific efficacy of various adjuvant therapies for paragangliomas still requires extensive clinical data to evaluate.

There is a family association between paraganglioma and the mitochondrial SDH gene, with 25% to 50% of cases being inherited. Paraganglioma risk is definitely raised by mutations in the SDH gene,^[16]^ with the highest rate of mutations in the B subunit,^[17]^ and mutations in the B subunit imply an increased risk of metastasis. Therefore, it has been proposed that all paraganglioma patients, as well as the immediate relatives of patients with gene abnormalities, undergo genetic testing.

In this case the tracheal bulge was significantly distorted by the tumor compression and the patient discovered a mediastinal mass by coincidence. However, the patient did not experience any discomfort, such as dyspnea or shortness of breath. Due to the lack of pathological diagnosis, the clinical diagnosis was considered to be hemangioma, and the location of the tumor was biased to the right side, so the right side open thoracotomy was performed without extracorporeal circulation, and the tumor envelope was seen to be intact during the operation, with no signs of tumor infiltration. The intraoperative bleeding was about 2400 mL, and the blood volume was supplemented with suspended red blood cells and plasma to stabilize the hemodynamics.

The biggest mistake in the treatment of this patient was the failure to perform preoperative vascular imaging and the lack of consideration for preoperative prophylactic embolization to prevent major intraoperative bleeding. This led to significant hemodynamic fluctuations during the surgery. Preoperative angiography is crucial for such mediastinal tumors that exhibit widespread enhancement on enhancement in order to evaluate the tumor’s vascular supply and, if required, carry out prophylactic embolization.

Paragangliomas generally have a good prognosis, but they have malignant potential and may manifest malignant behavior clinically even if the tumor is benign. Recurrence and metastasis may also occur after resection of paraganglioma, and the most common metastatic complements are lung, lymph nodes, and bone. Data from Erickson et al^[2]^ suggest that approximately 31% of patients with benign paraganglioma cannot be cured by surgical resection; according to Van Slycke et al,^[18]^ the average local recurrence rate of extra-adrenal paraganglioma at 5 years after surgery was 15 ± 7%, and the average local recurrence rate at 10 years was 23 ± 9%, the recurrence rate was 23 ± 9%. Paraganglioma patients must thus undergo long-term follow-up, and high-risk patients, such as those who are younger, have genetic problems, have big tumors, or both, should undergo annual follow-up for the rest of their lives.^[19]^

## Author contributions

**Conceptualization:** Shaopeng Xu, Jinchen Du, Linchong Ma, Qingxin Li.

**Data curation:** Shaopeng Xu, Lei Zou.

**Formal analysis:** Shaopeng Xu, Jinchen Du.

**Funding acquisition:** Gawei Hu.

**Supervision:** Gawei Hu, Qingxin Li.

**Validation:** Gawei Hu.

**Writing – original draft:** Shaopeng Xu, Linchong Ma.

**Writing – review & editing:** Gawei Hu, Qingxin Li.
